# A Review of the Recent Monkeypox Outbreak in 2022

**DOI:** 10.7759/cureus.27880

**Published:** 2022-08-11

**Authors:** Saleh Al-Gburi, Zainab Namuq

**Affiliations:** 1 Mosul Medical College, University of Mosul, Mosul, IRQ

**Keywords:** infectious disease, review article, orthopoxvirus, outbreak, monkeypox

## Abstract

The coincidence of the monkeypox outbreak with the coronavirus disease 2019 (COVID-19) pandemic raises a global concern about a potential new pandemic and the possible consequences. As the World Health Organization declared the international monkeypox outbreak a global emergency, there is apprehension about the complications and mortality of monkeypox infection.

The monkeypox virus is a zoonosis: a disease that is transmitted from animals to humans. It is an enveloped double-stranded DNA virus that belongs to the Orthopoxvirus genus. Human-to-human transmission occurs with close contact with respiratory secretions, sores on an infected person's skin, or contaminated items like clothing.

Monkeypox is endemic in regions of Africa, however, because of smallpox eradication and a decrease in vaccination efforts, this led to an outbreak in the United States of America in 2003 and a new world outbreak in 2022.

Most patients experience prodromal sickness with fever, malaise, and enlarged lymph nodes before developing a rash. In addition to skin lesions, individuals may also experience secondary skin and/or soft tissue infection, pneumonitis, ocular problems, and encephalitis. There is an increased risk of infection among men who have sex with men and human immunodeficiency virus infection (HIV) patients. The polymerase chain reaction is the gold standard for diagnosis. Management is usually supportive but some cases may require tecovirimat. This is a comprehensive review of monkeypox virus epidemiology, clinical features, and the most up-to-date, effective management and prevention.

## Introduction and background

The monkeypox virus causes a smallpox-like illness in humans. It is an enveloped double-stranded DNA virus that belongs to the Orthopoxvirus genus [1]. Rabbits and non-human primates are the main hosts of poxviruses, which can occasionally be transmitted to humans, enabling the incidence of human-to-human transmission [2].

Monkeypox is a zoonosis: a disease that is transmitted from animals to humans. When a person comes into contact with an infected animal, monkeypox can be transmitted to them. Avoiding unprotected contact with wild animals, especially those that are sick or dead, can lower the chances of contracting monkeypox from them (including their meat and blood) [3]. Any items containing animal meat or parts should be fully prepared before consumption in endemic nations where monkeypox is spread by animals [3].

Close contact with respiratory secretions, sores on an infected person's skin, or contaminated items like clothing and bedding can lead to human-to-human transmission. Although, extended face-to-face contact is often necessary for transmission by respiratory droplet particles. This puts close family members, household members, and other contacts of active patients at increased risk [4].

A monkeypox epidemic was verified in the United Kingdom (UK) on May 6, 2022. It was brought about by a British citizen who visited Nigeria, where the disease is widespread. On May 4, this traveler returned to the UK, bringing the outbreak's index case with them [5].

On Saturday 23/07/2022, the World Health Organization declared the international monkeypox outbreak a global emergency, the highest level of alert the WHO can issue [6]. This declaration raises public concern about the new pandemic and because the monkeypox outbreak coincides with the COVID-19 pandemic, there is concern about the severity and mortality of monkeypox cases. This is a review of monkeypox virus epidemiology, clinical features, and the most up-to-date effective management and prevention.

## Review

Epidemiology

The first incidence of the monkeypox virus was the outbreak of a pox-like disease in 1959 in monkeys hosted at a research center in Copenhagen, Denmark [7]. As the first human monkeypox virus case in medical history, a nine-month-old child from the Democratic Republic of the Congo (then known as the Republic of the Congo) was hospitalized at the Basankusu Hospital on September 1, 1970 [8]. Since then, there have been intermittent cases of monkeypox in people across that region, but no cases have been documented outside of Africa [9-11]. In 2003, the United States had the first monkeypox outbreak in the Western Hemisphere [12].

As of May 21, 2022, 92 cases have been verified globally, coming from 13 nations (the UK, Australia, Belgium, Canada, France, Germany, Italy, Netherlands, Portugal, Spain, Sweden, and the USA) where the monkeypox virus is not endemic [13]. On May 24, the United Arab Emirates (UAE) became the first Arab nation to disclose a case of infection [14].

A total of 3413 laboratory-confirmed cases and one death have been reported to WHO from 50 countries and territories in five WHO regions since January 1 up to June 22, 2022. Two thousand thirty-three out of 3413, or 86%, of the laboratory-verified cases, came from the WHO European Region. The African Region (73/3413, 2%), the Region of the Americas (381/3413, 11%), the Eastern Mediterranean Region (15/3413, 1%), and the Western Pacific Region (11/3413, 1%) are the other regions reporting cases. In the second quarter of 2022, Nigeria recorded one death [15].

Clinical signs

Most patients experience prodromal sickness with fever, malaise, and enlarged lymph nodes following a 10-14 day incubation period before developing a rash [16-17]. Clinically, smallpox and modified smallpox are similar to human monkeypox [18]. Since lymphadenopathy is seen in 90% of unvaccinated patients but is uncommon in smallpox, it is thought to be a key factor in differentiating monkeypox from smallpox [19]. In the submandibular, cervical, or inguinal areas, lymph nodes might swell [18].

Typically, the prodromal phase lasts one to three days before the classic maculopapular rash appears, The clinical progression is remarkably similar to that of typical smallpox lesions, and the mean diameter of the skin lesions is between 0.5 and 1 cm [19]. Lesions develop during the course of two to four weeks from macules to papules, vesicles, pustules, umbilication, scabbing, and desquamation [18].

**Figure 1:**
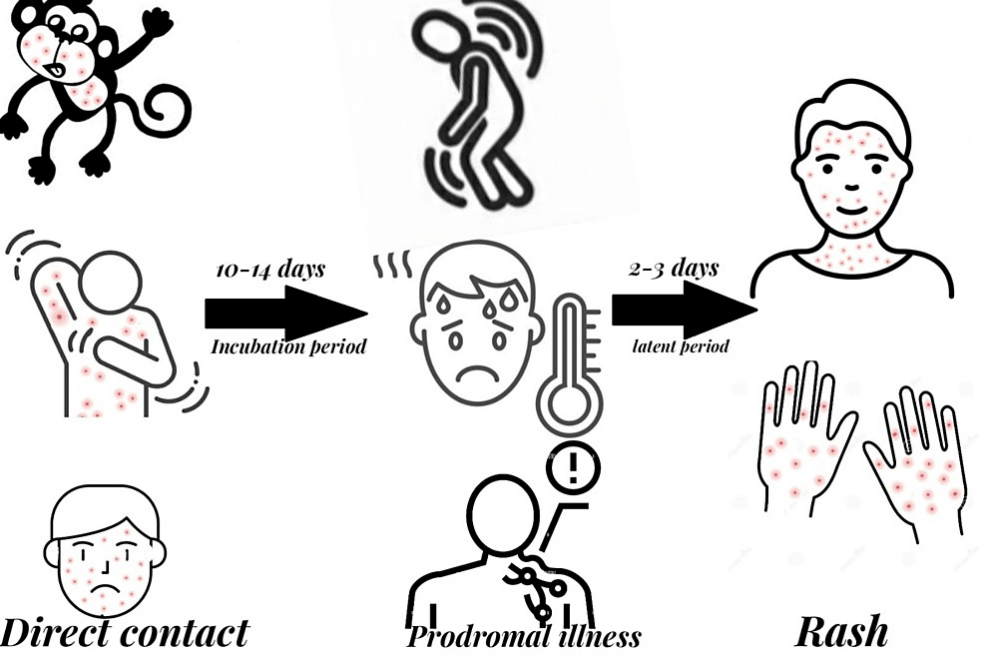
Clinical features of monkeypox infection Image credits: Saleh Al-Gburi, Zainab Namuq

In addition to skin lesions, individuals may also experience extracutaneous symptoms such as secondary skin and/or soft tissue infection, pneumonitis, ocular problems, and encephalitis [17]. The 10% mortality rate often occurs in the second week of the illness [20]. The hazards of monkeypox during pregnancy and how the virus can be transmitted to the fetus in the womb, the newborn during or after birth, or a nursing infant need more study. According to the knowledge that is currently available, getting monkeypox while pregnant can be harmful to the fetus [3]. The clinical features of monkeypox infection are shown in Figure 1.

Diagnosis

Monkeypox can be diagnosed using the clinical signs, but to distinguish this illness from those caused by other potential etiologies, laboratory confirmation is required [21]. Genetic, morphological, and immunological procedures are among the techniques utilized to confirm monkeypox in specimen analysis [1]. The polymerase chain reaction is the premier diagnostic test. The viral DNA within the lesion persists constantly for a long time if stored in a dark, rather cool environment, in addition to its great accuracy and sensitivity [22].

Unfortunately, a lot of these techniques are quite general and cannot distinguish between infection with the monkeypox virus and infection with other poxviruses. For instance, histologically, the lesions caused by monkeypox are comparable to those caused by other viral exanthems (such as those caused by cowpox, varicella zoster, and herpes simplex viruses) and feature ballooning keratinocyte degeneration, prominent spongiosis, dermal edema, and acute inflammation [23]. Orthopoxviruses and other orthopoxviruses react crossreactively in immunological testing. Even so, these tests could be helpful if there have been prior clues that point to the disease's origin [24].

Immunoglobulin M (IgM) is thought to be more useful in diagnosing recent infections, even while IgG alone cannot offer a conclusive diagnosis to a patient who has been exposed to the orthopoxvirus throughout his life through vaccination [25].

Management

The clinical course of monkeypox infection, fortunately, is often mild and self-limiting. Because of this, particular therapy is rarely warranted, and treatment is frequently supportive. Antipyretics for fever, analgesics for pain, and other forms of supportive therapy [3].

The first antiviral medication to enter clinical use was methisazone, but it is no longer in use since it was quite hazardous when given systemically [26]. A broad-spectrum antiviral medication called cidofovir has efficacy against a variety of DNA viruses, including MPXV [27], Although extensive testing on lab animals, cidofovir has not been utilized to treat orthopoxvirus infection in people [28-30]. Other drugs have demonstrated antipoxvirus efficacy in vitro or in a variety of small animal models [31]. The European Medicines Agency authorized tecovirimat, an antiviral created to treat smallpox, in January 2022 to treat monkeypox [3].

Prevention

The final consequence of the smallpox eradication campaign was the complete elimination of the disease worldwide. Monkeypox cannot be eradicated, regrettably, because a reservoir of animals is present [19]. But inoculation with the vaccinia virus (smallpox vaccine) is quite effective at preventing monkeypox infection [9,32-33].

People who are at risk should get vaccinated, according to certain nations. Smallpox vaccines may also be helpful for monkeypox. Only those who are at risk, for instance, someone who has had intimate contact with someone who has monkeypox, should be given the vaccine. At this time, widespread immunization is not advised [3]. The European Medicines Agency has approved the smallpox vaccine, IMVANEX, for use against monkeypox [34].

In any hospitalized patient with a broad vesicular rash of uncertain cause in whom monkeypox and smallpox are included in the differential diagnosis, standard, contact, droplet, and airborne precautions should be undertaken. Close contacts should also be kept an eye on [21]. In the hospital, patients should be segregated in rooms with negative pressure and the medical personnel should take proper contact and droplet precautions [35]. Before interacting with these patients, medical professionals should put on properly fitted N95 masks, gloves, and eye protection. This should continue until the lesions have crusted and the scabs have come off [36]. According to the data, monkeypox is less contagious among humans than smallpox, and the longest chain of infected persons is six cases [22]. A summary of monkeypox virus case reports published in 2022 is given in Table 1.

**Table 1:** A summary of monkeypox virus case reports published in 2022

Title of the case report	Age	Sex	Country	History of travel	Animal or human contact	Sexual status	Sexually transmitted infection	Human immunodeficiency virus	Systemic symptoms	Systemic symptoms - rash duration	Localization of lesion
Epidemiological, clinical, and virological characteristics of four cases of monkeypox support transmission through sexual contact, Italy, May 2022 [37].	30s	Male	Italy	Yes, Gran Canary island	Sexual contact with human	Men sex with men	Hepatitis C, syphilis	Positive	No	not applicable	Genital, thorax, and calf area
Epidemiological, clinical, and virological characteristics of four cases of monkeypox support transmission through sexual contact, Italy, May 2022 [37].	30s	Male	Italy	Yes, Gran Canary island	Sexual contact with human	Men sex with men	Syphilis	Negative	Fever	3	Anal, back, legs, and foot sole
Epidemiological, clinical, and virological characteristics of four cases of monkeypox support transmission through sexual contact, Italy, May 2022 [37].	30s	Male	Italy	Yes, Gran Canary island	Sexual contact with human	Men sex with men	Syphilis, hepatitis B	Positive	Fever	3	Anal, head, thorax, legs, arms, hand, and genital area
Epidemiological, clinical, and virological characteristics of four cases of monkeypox support transmission through sexual contact, Italy, May 2022 [37].	30s	Male	Italy	Yes.	Sexual contact with human	Men sex with men	Hepatitis A	Negative	Myalgia	2	Genital and pubic area
Monkeypox infection presenting as genital rash, Australia, May 2022 [38].	30s	Male	Australia	Yes, Europe	Sexual contact with human	Men sex with men	not applicable	Positive	Fever, malaise lymphadenopathy	2	Trunk, face, and limbs
Imported Monkeypox from an international traveler, Maryland, USA, 2021 [39].	28 years	Male	USA	Yes, Nige­ria	not applicable	No.	not applicable	not applicable	Lymphadenopathy		Face, neck, and arms
The first case of monkeypox in the Republic of Korea [40].	34 years	Male	Republic of Korea	Yes, Germany	None.	Bisexual	None	None	Fever, sore throat, and lymphadenopathy	3	Face, back, and lower abdomen
Monkeypox genital Lesions [41].	31 years	Male	not applicable	not applicable	Sexual contact with human	Men sex with men	Negative	Positive	Without prodromal symptoms	not applicable	Painless anogenital lesions
Monkeypox infection in a developed country: A Case Report [42].	30s	Male	Israel	None	Sexual contact with human	Men sex with men	Condyloma Acuminatum	Negative	Fever, fatigue, and muscle aching	1 day	Anal and perianal area, neck, and trunk
Monkeypox infection in a developed country: A Case Report [42].	30s	Male	Israel	Yes, Europe	Sexual contact with human	Men sex with men	Condyloma Acuminatum	Positive	Malaise, dysuria, penile pruritus, and unilateral inguinal lymphadenopathy	2 days	Penis and then all over the body sparing the face
Human monkeypox coinfection with acute HIV: an exuberant presentation [43].	24 years	Male	Portugal	not applicable	Sexual contact with human	Men sex with men	negative	Positive	Fatigue, fever, and anal pain appear with the rash	None	Perianal, genitalia, and then trunk

## Conclusions

The introduction of a disease like monkeypox into a new, previously disease-free region of the world can provoke a global concern of potential pandemic. Social distancing and contact tracing are fundamental because human-to-human transmission most commonly occurs via respiratory droplets or direct contact with an infected individual.

Given the sharp rise in positive cases, monkeypox's public health implications testing should be made more widely available, and awareness of the sickness caused by human monkeypox should also rise. The necessity for self-quarantine and the mechanisms of transmission must be explained to patients who are suspected or have been diagnosed. Increasing public awareness about the mode of transmission, clinical symptoms, and prevention methods will limit the progression of this disease. Recommendation for vaccination especially among high-risk individuals (men who have sex with men and HIV patients) will prevent the evolution of disease throughout the world. Monkeypox's early clinical manifestations may resemble those of other sexually transmitted infections, including syphilis, herpes, or lymphogranuloma venereum. In patients with epidemiologic risk factors for monkeypox, a thorough history should be conducted together with a physical examination. Appropriate specimens should also be gathered from lesions and tested for the monkeypox virus.

## References

[1] Alakunle E, Moens U, Nchinda G, Okeke MI (2020). Monkeypox virus in Nigeria: infection biology, epidemiology, and evolution. Viruses.

[2] Haller SL, Peng C, McFadden G, Rothenburg S (2014). Poxviruses and the evolution of host range and virulence. Infect Genet Evol.

[3] (2022). Monkeypox. http://www.who.int/news-room/questions-and-answers/item/monkeypox?gclid=Cj0KCQjwof6WBhD4ARIsAOi65ajlacTIz2_Y--hjACfn.

[4] Brown K, Leggat PA (2016). Human monkeypox: current state of knowledge and implications for the future. Trop Med Infect Dis.

[5] (2022). Monkeypox - United Kingdom of Great Britain and Northern Ireland. https://www.who.int/emergencies/disease-outbreak-news/item/2022-DON383.

[6] (2022). Second meeting of the International Health Regulations (2005) (IHR) Emergency Committee regarding the multi-country outbreak of monkeypox. http://www.who.int/news/item/23-07-2022-second-meeting-of-the-international-health-regulations-(2005)-(ihr)-emergency-committee-regarding-the-multi-country-outbreak-of-monkeypox.

[7] Magnus Pv, Andersen EK, Petersen KB, Birch‐Andersen A (1959). A pox‐like disease in cynomolgus monkeys. Acta Pathol Microbiol Scand.

[8] Breman JG, Kalisa-Ruti Kalisa-Ruti, Steniowski MV, Zanotto E, Gromyko AI, Arita I (1980). Human monkeypox, 1970-79. Bull World Health Organ.

[9] Jezek Z, Marennikova SS, Mutumbo M, Nakano JH, Paluku KM, Szczeniowski M (1986). Human monkeypox: a study of 2,510 contacts of 214 patients. J Infect Dis.

[10] Mukinda V, Mwema G (1997). Re-emergence of human monkeypox in Zaire in 1996. Lancet.

[11] Hutin YJ, Williams RJ, Malfait P (2001). Outbreak of human monkeypox, Democratic Republic of Congo, 1996 to 1997. Emerg Infect Dis.

[12] Reed KD, Melski JW, Graham MB (2004). The detection of monkeypox in humans in the Western Hemisphere. N Engl J Med.

[13] (2022). Multi-country monkeypox outbreak in non-endemic countries: update. https://www.who.int/emergencies/disease-outbreak-news/item/2022-DON388.

[14] Monkeypox: Israel (2022). Monkeypox: Israel, Switzerland and Austria confirm cases. http://www.bbc.com/news/health-61540474.

[15] (2022). Multi-country monkeypox outbreak: situation update. http://www.who.int/emergencies/disease-outbreak-news/item/2022-DON396#:~:text=Since%201%20January%20and%20as,new%20countries%20have%20reported%20cases..

[16] Jezek Z, Fenner F (1988). Human Monkeypox. https://www.karger.com/Book/Home/221547.

[17] Di Giulio DB, Eckburg PB (2004). Human monkeypox: an emerging zoonosis. Lancet Infect Dis.

[18] Fenner F, Henderson D, Arita I, Jezek Z, Ladnyi I (1988). Human monkeypox and other poxvirus infections of man. Smallpox and its Eradication Geneva, Switzerland: World Health Organization.

[19] Nalca A, Rimoin AW, Bavari S, Whitehouse CA (2005). Reemergence of monkeypox: prevalence, diagnostics, and countermeasures. Clin Infect Dis.

[20] Frey SE, Belshe RB (2004). Poxvirus zoonoses—putting pocks into context. N Engl J Med.

[21] Isaacs SN (2022). Monkeypox. Literature review current through: Apr.

[22] McCollum AM, Damon IK (2014). Human monkeypox. Clin Infect Dis.

[23] Bayer-Garner IB (2005). Monkeypox virus: histologic, immunohistochemical and electron-microscopic findings. J Cutan Pathol.

[24] Hraib M, Jouni S, Albitar MM, Alaidi S, Alshehabi Z (2022). The outbreak of monkeypox 2022: an overview. Ann Med Surg (Lond).

[25] Cho CT, Wenner HA (1973). Monkeypox virus. Bacteriol Rev.

[26] Plaut M, Tinkle SS (2003). Risks of smallpox vaccination: 200 years after Jenner. J Allergy Clin Immunol.

[27] De Clercq E (2002). Cidofovir in the treatment of poxvirus infections. Antivir Res.

[28] Smee DF, Bailey KW, Wong M-H, Sidwell RW (2000). Intranasal treatment of cowpox virus respiratory infections in mice with cidofovir. Antivir Res.

[29] Bray M, Martinez M, Smee DF, Kefauver D, Thompson E, Huggins JW (2000). Cidofovir protects mice against lethal aerosol or intranasal cowpox virus challenge. J Infect Dis.

[30] Smee DF, Bailey KW, Wong M-H, Sidwell RW (2001). Effects of cidofovir on the pathogenesis of a lethal vaccinia virus respiratory infection in mice. Antivir Res.

[31] Baker RO, Bray M, Huggins JW (2003). Potential antiviral therapeutics for smallpox, monkeypox and other orthopoxvirus infections. Antiviral research.

[32] Fine PE, Jezek Z, Grab B, Dixon H (1988). The transmission potential of monkeypox virus in human populations. Int J Epidemiol.

[33] Arita I, Jezek Z, Khodakevich L, Ruti K (1985). Human monkeypox: a newly emerged orthopoxvirus zoonosis in the tropical rain forests of Africa. Am J Trop Med Hyg.

[34] (2022). Monkeypox vaccine from Bavarian Nordic wins EU approval. http://www.reuters.com/business/healthcare-pharmaceuticals/bavarian-nordic-monkeypox-vaccine-wins-eu-approval-2022-07-25/.

[35] Moore MJ, Rathish B, Zahra F (2022). Monkeypox. http://pubmed.ncbi.nlm.nih.gov/34662033/.

[36] (2022). Multi-country monkeypox outbreak in non-endemic countries. https://www.who.int/emergencies/disease-outbreak-news/item/2022-DON385.

[37] Antinori A, Mazzotta V, Vita S (2022). Epidemiological, clinical and virological characteristics of four cases of monkeypox support transmission through sexual contact, Italy, May 2022. Euro Surveill.

[38] Hammerschlag Y, MacLeod G, Papadakis G (2022). Monkeypox infection presenting as genital rash, Australia, May 2022. Euro Surveill.

[39] Costello V, Sowash M, Gaur A, Cardis M, Pasieka H, Wortmann G, Ramdeen S (2022). Imported monkeypox from international traveler, Maryland, USA, 2021. Emerg Infect Dis.

[40] Jang YR, Lee M, Shin H (2022). The first case of monkeypox in the Republic of Korea. J Korean Med Sci.

[41] Patrocinio-Jesus R, Peruzzu F (2022). Monkeypox genital lesions. N Engl J Med.

[42] Patalon T, Perez G, Melamed G, Wolf T, Gazit S (2022). Monkeypox infection in a developed country: a case report [Preprint]. Research Square.

[43] de Sousa D, Patrocínio J, Frade J, Correia C, Borges-Costa J, Filipe P (2022). Human monkeypox coinfection with acute HIV: an exuberant presentation. Int J STD AIDS.

